# The Efficacy and Safety of Pulmonary Vasodilators in Pediatric Pulmonary Hypertension (PH): A Systematic Review and Meta-analysis

**DOI:** 10.3389/fphar.2021.668902

**Published:** 2021-04-23

**Authors:** Tingting Shu, Huaqiao Chen, Lu Wang, Wuwan Wang, Panpan Feng, Rui Xiang, Li Wen, Wei Huang

**Affiliations:** Department of Cardiology, The First Affiliated Hospital of Chongqing Medical University, Chongqing, China

**Keywords:** pediatric pulmonary hypertension, efficacy, safety, meta-analysis, pulmonary vasodilators

## Abstract

**Background:** We performed a meta-analysis to evaluate the efficacy and safety of pulmonary vasodilators in pediatric pulmonary hypertension (PH) patients.

**Methods:** We searched electronic databases including PubMed, EMBASE, and the Cochrane Library up to May 2020, and conducted a subgroup analysis for pulmonary vasodilators or underlying disease.

**Results:** Fifteen studies with 719 pediatric PH patients were included in the meta-analysis. Adverse events did not differ (*p* = 0.11, *I* ^2^ = 15%) between the pulmonary vasodilators group and the control group, neither in the subgroups. In total, compared with the control group treatment, pulmonary vasodilators significantly decreased the mortality (*p* = 0.002), mean pulmonary artery pressure (mPAP, *p* = 0.02), and mechanical ventilation duration (*p* = 0.03), also improved the oxygenation index (OI, *p* = 0.01). In the persistent pulmonary hypertension of the newborn (PPHN) subgroup, phosphodiesterase type 5 inhibitors (PDE5i) significantly reduced mortality (*p* = 0.03), OI (*p* = 0.007) and mechanical ventilation duration (*p* = 0.004). Administration of endothelin receptor antagonists (ERAs) improved OI (*p* = 0.04) and mechanical ventilation duration (*p* < 0.00001) in PPHN. We also found that in the pediatric pulmonary arterial hypertension (PPAH) subgroup, mPAP was pronouncedly declined with ERAs (*p* = 0.006). Systolic pulmonary artery pressure (sPAP, *p* < 0.0001) and pulmonary arterial/aortic pressure (PA/AO, *p* < 0.00001) were significantly relieved with PDE5i, partial pressure of arterial oxygen (PaO_2_) was improved with prostacyclin in postoperative PH (POPH) subgroup (*p* = 0.001). Compared with the control group, pulmonary vasodilators could significantly decrease PA/AO pressure (*p* < 0.00001) and OI (*p* < 0.00001) in the short-term (duration <7 days) follow-up subgroup, improve mPAP (*p* = 0.03) and PaO_2_ (*p* = 0.01) in the mid-term (7–30 days) follow-up subgroup, also decrease mortality, mPAP (*p* = 0.0001), PA/AO pressure (*p* = 0.0007), duration of mechanical ventilation (*p* = 0.004), and ICU stay (*p* < 0.00001) in the long-term follow subgroup (>30 days).

**Conclusion:** Pulmonary vasodilators decrease the mortality in pediatric PH patients, improve the respiratory and hemodynamic parameters, reduce the mechanical ventilation duration.

## Introduction

Pediatric pulmonary hypertension (PH) is associated with severe morbidity and mortality (Berger et al., 2011; Frantz et al., 2013; Hoeper et al., 2013). Similar to adult PH, the definition of pediatric PH was updated in The Pediatric Task Force of the 6th World Symposium on Pulmonary Hypertension (WSPH) in Nice, France (2018): mean pulmonary artery pressure (mPAP) > 20 mmHg, normal pulmonary capillary wedge pressure (mPCWP) ≤ 15 mmHg, and pulmonary vascular resistance (PVR) > 3 WU (ten Harkel et al., 2019). According to the nationwide Netherlands PH Service (Berger et al., 2011) the yearly incidence rate for all PH diagnoses is 63.7 cases per million children. Persistent pulmonary hypertension of the newborn (PPHN) and cardiogenic PH had the highest incidence rates: 30.1 and 21.9 cases per million children, respectively. Progressive PH had an annual incidence rate of 3.0 cases per million children. For idiopathic pulmonary artery hypertension (iPAH) and PH associated with congenital heart disease (PH-CHD), the incidence was even lower: 0.7 and 2.2 cases per million. In addition, the subgroup of patients with postoperative PH (POPH) following CHD repair occurs in 21.9 cases per million and is one of the most common forms of PAH in children (Frantz et al., 2013; Hoeper et al., 2013).

There are three main options to treat PH in the clinic: endothelin, endothelin receptor antagonist, and prostacyclin (Galiè et al., 2015). Most treatment strategies for pediatric PH patients are based on adult clinical trial data and clinical experience. For example, Bosentan, as a dual endothelin receptor antagonist (ERA), which was approved by the FDA in September 2017 for pediatric use in patients over 3 years, exhibited positive efficacy in several pediatric PH trials (Gibbs and Ismail, 2012; PAN and KONG, 2016; Steinhorn et al., 2016). However, it has been reported that chronic use of bosentan may lead to elevated liver aminotransferases, also cause liver cirrhosis. Another drug, sildenafil can improve peak oxygen consumption, functional category, and hemodynamics in pediatric PH patients based on the double-blind, placebo-controlled Sildenafil in Treatment-naive Children, Aged 1–17 years, With Pulmonary Arterial Hypertension (STARTS-1) and Long-Term Survival With Oral Sildenafil Monotherapy in Treatment-Naive Pediatric Pulmonary Arterial Hypertension (STARTS-2) trials (Cornelisse et al., 2012; Szatmari et al., 2014). However, the STARTS-2 trial reported higher mortality in the high-dose sildenafil group (80 mg) (Szatmari et al., 2014). The FDA announced in 2014 that sildenafil could be considered when the benefits of this drug treatment may exceed its potential risk to each patient, but high-dose sildenafil needs to be warned (Layton and Bavdekar, 2015). The prostacyclin analog treprostinil was approved by FDA for adult PH patients in 2002, and it is also used in the clinic for pediatric patients who cannot be improved by other treatments and showed good efficacy (Rosenzweig et al., 1999). It is worth noting that the use of prostacyclin analogs may be associated with an increased risk of bleeding in patients (Wade et al., 2003). The choice of pulmonary vasodilators for different types of pediatric PH patients is currently controversial. Therefore, we conducted a meta-analysis to comprehensively evaluate the efficacy and safety of pulmonary vasodilators in pediatric patients with PH, and we also conducted a further subgroup analysis to compare the efficacy and safety of different subgroups of pulmonary vasodilators in the treatment of subgroups of pediatric PH.

## Methods

### Search Strategy and Data Collecting

This meta-analysis was performed in accordance with the Preferred Reporting Items for Systematic Reviews and Meta-Analyses (PRISMA) statement (Shamseer et al., 2015). The electronic databases included PubMed, EMBASE, and the Cochrane Library up to May of 2020, and the searches were conducted by Me-SH terms and keywords. The keywords were searched in various combinations using the Boolean operators “AND” and “OR”. Search expressions are shown in Supplementary Table S1. We manually searched clinical trial registries (clinicaltrials.gov) and the International Clinical Trials Registry Platform (www.whoint/ictrp/search/en/). We also searched the reference lists of all the selected articles and searched the studies published in peer-reviewed journals for additional related publications. Both the electronic search and manual search were performed independently by two authors (TT Shu and HQ Chen).

### Study Inclusion and Exclusion Criteria

The inclusion criteria were as follows: 1) studies that were randomized controlled trials; 2) studies in which the participants comprised newborns, infants, and children (postnatal to 18 years) diagnosed with PPHN, POPH or PPAH (idiopathic PH, PH associated with connective tissue disease or congenital heart disease without repair surgery); 3) studies in which the diagnosis of PH was based on clinical findings with echocardiographic or right heart catheter confirmation; 4) studies comparison of mono-therapy with pulmonary vasodilators vs. placebo or no treatment; 5) the outcomes were reported in both pulmonary vasodilators group and control group. Animal therapy trials and preclinical trials were excluded. The duplicated studies were removed and the study with the largest sample size from the same institute was remained. Two independent researchers (TT Shu and HQ Chen) selected studies for inclusion by scanning the title, abstract, and full text.

### Data Extraction and Quality Assessment

The following data were extracted: 1) study characteristics (first author, publication year, and participant sample size), 2) pharmacotherapy intervention, and 3) study results data. The review authors compared the results and resolved any discrepancies. One review author imported the extracted data into RevMan 5.3 software, and another author checked the imported data. If relevant articles were identified that provided insufficient data or did not provide relevant data for review, then the authors obtained the data from the authors of the relevant study by email.

The primary outcomes were defined as mortality and adverse events. Safety was evaluated by the adverse events. Adverse events were defined as headache, gastrointestinal upset (including nausea or vomiting), respiratory symptoms, abnormal hepatic function, hematological disorders (including anemia, red blood cell transfusion, thrombocytopenia, or epistaxis), and PH crisis. Secondary outcomes included the changes from baseline to follow-up: 1) respiratory and hemodynamic parameters, including oxygenation index (OI = fraction of inspired oxygen × mean airway pressure/arterial oxygen pressure), partial pressure of oxygen (PaO_2_), blood oxygen saturation (SpO_2_), mPAP, systolic PAP (sPAP), mean pulmonary arterial/aortic (PA/AO) pressures; 2) duration of mechanical ventilation; and 3) intensive care unit (ICU) stay (GalièGhersi et al., 2015).

Trials comparing the same type of pulmonary vasodilators but at different therapeutic doses (fixed or flexible-dose) and different treatment durations were grouped in the same node in the meta-analysis. The studies were classified into followed-up duration subgroups: short-term follow-up (<7 days), mid-term follow-up (7–30 days), long-term follow-up (>30 days). The included studies were analyzed using the RevMan 5.3 software, and we computed 95% confidence intervals (CIs) of mean differences (MDs) for the continuous outcome data and risk ratios (RRs) with a 95% CI for dichotomous outcome data. Values of *p* < 0.05 indicated statistical significance. The chi^2^ test was conducted on the research effect size to evaluate heterogeneity. We followed the CNRG recommendations by using the following criteria to describe the heterogeneity: < 25% no heterogeneity, 25–49% low heterogeneity, 50–74% moderate heterogeneity, and ≥75% high heterogeneity. When the research effect size was homogeneous, i.e., I^2^ greater than 50%, data were reanalyzed using a random-effects model, the Mantel-Haenszel method. If there was considerable bias, we explored the effect of bias by conducting a sensitivity analysis. The sensitivity analysis of results was performed by excluding low-quality studies or subgroups with different types of drugs or underlying diseases.

To select high-quality studies, we independently rated the quality of each retrieved study by the Cochrane Risk of Bias tool in the RevMan 5.3 software (Gibbs et al., 2011). The GRADE approach was utilized to assess the quality of evidence for the following outcomes: mortality, adverse events, respiratory and hemodynamic parameters (OI, PaO_2_, SpO_2_, mPAP, sPAP, PA/AO), mechanical ventilation duration and ICU stay. When the following five factors were included, the level of evidence was downgraded one level for serious or two levels for very serious limitations: risk of bias, consistency, directness, precision, and publication bias. According to the GRADE approach, the evidence was divided into four levels: high, moderate, low, and very low. The higher the level of evidence, the higher the confidence of this study in evaluating treatment effects.

## Results

### Study Characteristics

The search strategy revealed a total of 499 studies, of which fifteen studies with 719 pediatric PH patients met the inclusion criteria and were included in the analysis. The flow diagram of document selection for this analysis is shown in Supplementary Figure S2 . The follow-up duration ranged from 24 h to 15 months. The main characteristics of the included studies are shown in Table 1. Among the fifteen included studies, three had an ERA group and control group (Gibbs and Ismail, 2012; Pan and Kong, 2016; Steinhorn et al., 2016), nine had PDE5i and control groups (Juni et al., 2006; Wessel et al., 2006; Peiravian et al., 2007; Kolaee et al., 2009; Vargas-Origel et al., 2010; Fraisse et al., 2011; Uslu et al., 2011; Cornelisse et al., 2012; Sharma et al., 2015), and three had PGI_2_ treatment and control groups (Takahashi et al., 2003; Xu et al., 2015; Onan et al., 2016). Based on subgroups of pediatric PH patients, there were seven studies of PPHN (Juni et al., 2006; Wessel et al., 2006; Kolaee et al., 2009; Vargas-Origel et al., 2010; Uslu et al., 2011; Gibbs and Ismail, 2012; Steinhorn et al., 2016), two studies of PPAH (Cornelisse et al., 2012; PAN and KONG, 2016), and six studies of POPH (Takahashi et al., 2003; Peiravian et al., 2007; Fraisse et al., 2011; Sharma et al., 2015; Xu et al., 2015; Onan et al., 2016). A list of pulmonary vasodilator subgroups used in pediatric PH patients is shown in Table 2.

**TABLE 1:** The characteristics of the included studies.

Included trials, Year	N	Gender, N (Male/Female)	Age (E/C)	Weight (E/C, kg)	Intervention			Diagnosis	Follow-up	Measurement method
					Drug	Contrast	Drug dosages			
Uslu S, 2011	65	36/29	38.3/38.5 W	3.3/3.3	Sildenafil	MgSO_4_	0.5 mg/kg q6h	PPHN	14 M	Catheter
Baquero H, 2006	13	7/6	38.4/37.2 W	2.8/2.7	Sildenafil	Placebo	1 mg/kg q6h	PPHN	12 M	Catheter
Qiu gang, 2009	30	—	38.3/39.0 W	3.1/3.1	Sildenafil	Tolazoline	1 mg/kg, q4-8 h	PPHN	7 D	UCG
Vargas-origel A, 2009	51	22/29	37.8/38.8 W	3.0/3.0	Sildenafil	Placebo	3 mg/kg q6h	PPHN	25 H	UCG
Herrea TR, 2006	24	11/13	37.0/36.2 W	2.7/2.7	Sildenafil	Distilled water	1 mg/kg	PPHN	72 H	Catheter
Fraisse A, 2011	17	9/8	3.8 Y	18.6	Sildenafil	Placebo	Targeted concentration^c^	POPH	28 D	Catheter
Peiravian F, 2007	42	24/18	5.6/4.0 Y	14.3/12.9	Sildenafil	Placebo	0.3 mg/kg q3h	POPH	24 H	Catheter
Sharma VK, 2015	46	25/21	3.2/3.4 Y	12.6/12.8	Sildenafil	Placebo	0.5 mg/kg bid	POPH	16 W	Catheter
Barst RJ, 2011	234	89/145	1–17 Y^a^	17/17^b^	Sildenafil	Placebo	Targeted concentration^d^	PPAH	16 W	Catheter
Mohamed WA, 2012	47	26/21	39.7/38.8 W	3.5/3.5	Bosentan	Placebo	1 mg/kg bid	PPHN	6 M	UCG
Steinhorn RH, 2016	21	6/15	39.2/38.6 W	3.4/3.2	Bosentan	Placebo	2 mg/kg bid	PPHN	12 M	UCG
Yanyan pan, 2016	60	25/35	1.5 M	—	Bosentan	Captopril	2 mg/(kg·d) bid	PPAH	8 W	UCG
Onan IS, 2016	27	13/14	7.8/5.8 M	5.6/5.2	Iloprost	Placebo	2.0 ng/kg/min^e^	POPH	30 D	Catheters
Zhuoming xu, 2015	22	17/5	28.0/12.8 M	9.2/5.9	Iloprost	Placebo	50 ng/kg/min^f^	POPH	<30 D	UCG
Kazuhiro T, 2003	20	13/7	4.6/11.0 Y	—	Beraprost	Placebo	Beginning with 1ug/kg	POPH	15 M	Catheters

N = number, E = Experimental group, C = control group, M = months, W = weeks, D = days, H = hours, AE = adverse events, mPVRi = mean pulmonary vascular resistance index, q6h = every 6 h, q4-8 h = every 4–8 h, UCG = Ultrasonic cardiogram, PPHN = Persistent Pulmonary Hypertension of the Newborn, POPH = Pediatric Postoperative Pulmonary Hypertension, PPAH = Pediatric Pulmonary artery Hypertension.

a Age range.

b Body mass index.

c Targeted concentration: IV sildenafil to achieve target sildenafil plasma concentrations of approximately 40, 120, and 360 ng/ml in the low-, medium-, and high-dose groups, respectively.

d Targeted concentration: oral sildenafil achieve maximum plasma concentrations of 47, 140, and 373 ng/ml, respectively.

e Iloprost trometamol infusion (2.0 ng/kg/min) (BerliMed S.A. Madrid, Spain) was delivered intravenously after weaning from cardiopulmonary bypass. Intravenous iloprost was started in the operation theater and was delivered continuously during the postoperative intensive care unit (ICU) follow-up.

f Inhalation for 10 min q2h for 2 days.

**TABLE 2:** List of studies on pulmonary vasodilators for the treatment of different types of pediatric pulmonary hypertension.

Pulmonary vasodilators	Patients	Included studies
**PDE5i**	PPHN	Uslu S, 2011; baquero H, 2006; qiu gang, 2009; vargas-origel A, 2009; herrea TR, 2006
	POPH	Fraisse A, 2011; peiravian F, 2007; sharma VK, 2015
	PPAH	Barst RJ, 2011
**ERAs**	PPHN	Mohamed WA, 2012; steinhorn RH, 2016
	PAPH	Yanyan pan, 2016
**PGI**_**2**_	POPH	Onan IS, 2016; zhuoming xu, 2015; kazuhiro T, 2003

PPAH in this study include: IPAH, HPAH, PAH associated with connective tissue disease or congenital heart disease. PDE5i: phosphodiesterase type 5 inhibitor; ERAs: endothelin receptor antagonists; PGI_2_: Prostacyclin. Other abbreviations are declared as Table 1.

### Risk of Bias in the Included Studies

After excluding low-quality studies, the included studies were multicenter, double-blind, placebo-controlled trials. In the evaluation of attrition bias, 7 studies reported high risks, which accounted for more than 25%. The high-risk proportions of the remaining various biases were all less than 25%. The included studies in the present study were high quality. The details and a summary of the risk of bias in the included studies are presented in Supplementary Figures S3,S4.

### Overall Analysis of Pulmonary Vasodilators in Pediatric PH

#### Primary Outcomes

**FIGURE 1:**
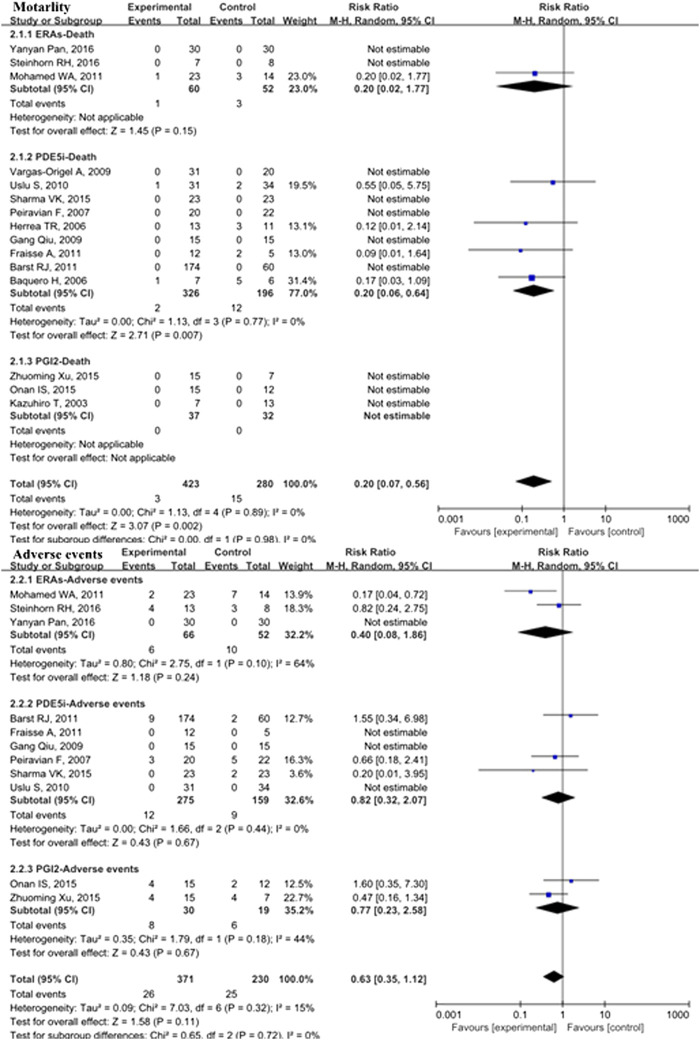
Mortality and adverse events in pediatric PH patients treated with pulmonary vasodilators. Abbreviations ERAs, endothelin receptor antagonists; PDE5i, phosphodiesterase type 5 inhibitors; PGI2, prostacyclins; PPHN, persistent pulmonary hypertension of the newborn; POPH, pediatric postoperative pulmonary hypertension; PPAH, pediatric pulmonary arterial hypertension.

**FIGURE 2:**
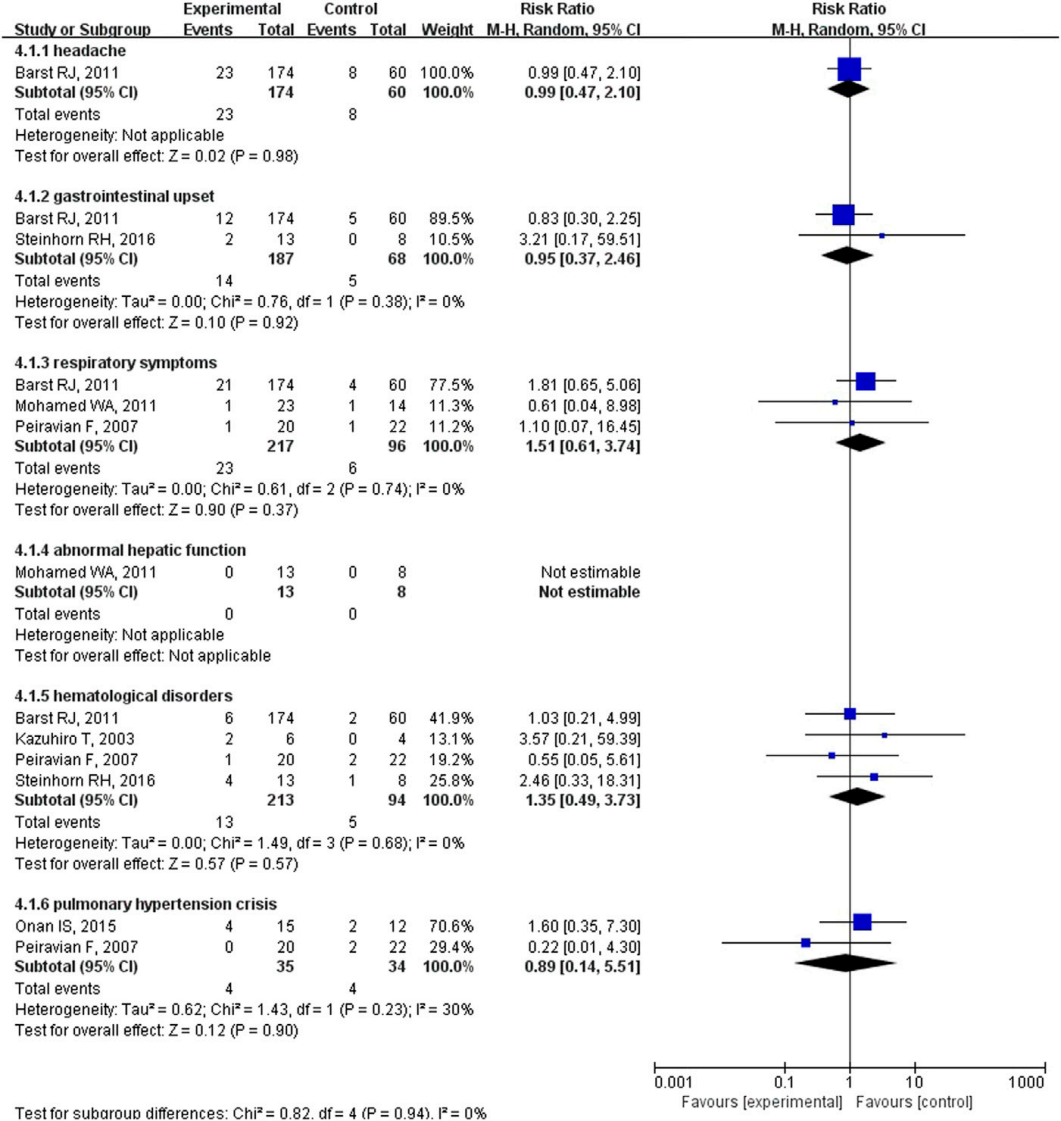
The incidence of adverse events in pediatric PH patients treated with pulmonary vasodilators comparing to the control group.

Fifteen studies with 703 patients evaluated pulmonary vasodilators and reported mortality. These studies showed that the mortality rate in the pulmonary vasodilator group (*n* = 423) was significantly lower (RR: 0.20; 95% CI: 0.07 to 0.56; *p* = 0.002; *I* ^2^ = 0%; Figure 1) than that in the control group (*n* = 280), equating to an absolute risk difference of −0.03 (95% CI, −0.07 to 0.02). Eleven trials with 601 patients were evaluated adverse events and no significant difference was observed (RR: 0.63; 95% CI: 0.35 to 1.12; *p* = 0.11, *I* ^2^ = 15%; Figure 1) between the pulmonary vasodilator group (*n* = 26) and the control group (*n* = 25), equating to an absolute risk difference of −0.02 (95% CI, −0.08 to 0.03). The incidence of headache (13.2 vs. 13.3%, *p* = 0.98, Figure 2), gastrointestinal upset (7.5 vs. 7.4%, *p* = 0.92), respiratory symptoms (10.6 vs. 6.3%, *p* = 0.37), abnormal hepatic function (0 vs. 0%, *p* = not applicable), hematological disorders (6.1 vs. 5.3%, *p* = 0.57), and PH crisis (11.4 vs. 11.8%, *p* = 0.90) in pulmonary vasodilator group was similar to the control group.

#### Secondary Outcomes

**FIGURE 3:**
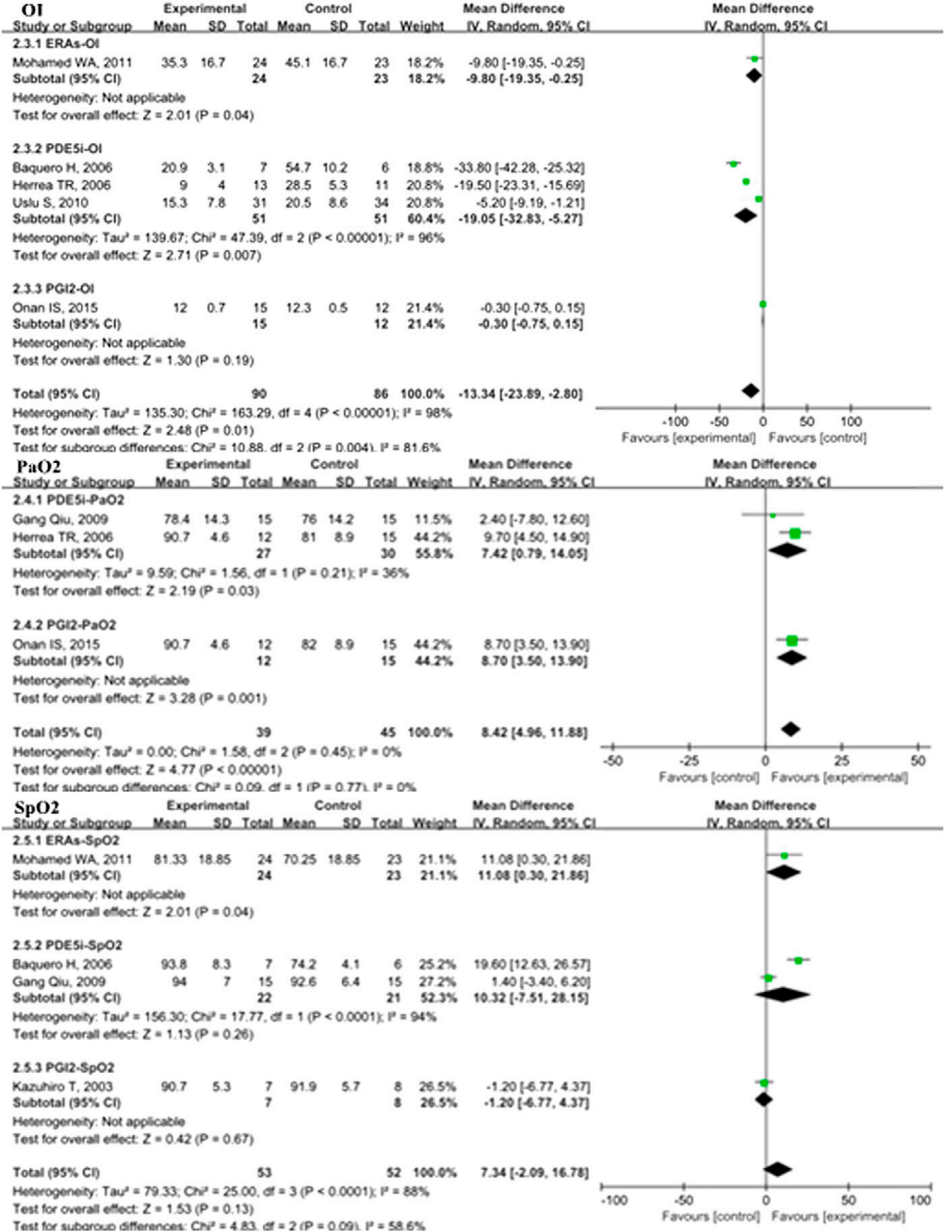
OI, PaO2, and SpO_2_ in pediatric PH patients treated with pulmonary vasodilators. Abbreviations: OI, oxygenation index; PaO2, partial pressure of arterial oxygen; SpO2, pulse oxygen saturation. Other abbreviations are defined in Figure 1.

**FIGURE 4:**
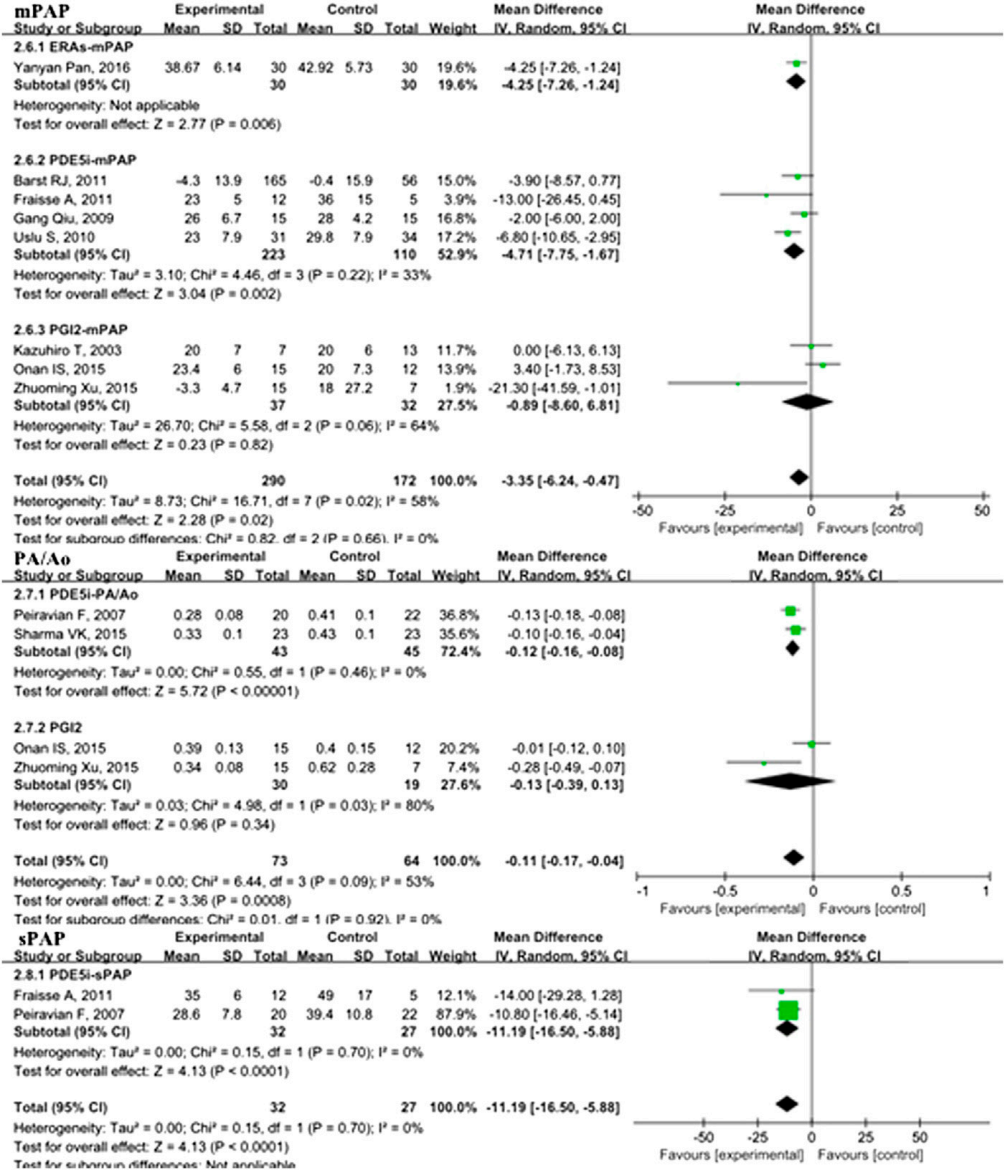
mPAP, PA/Ao, and sPAP in pediatric PH patients treated with pulmonary vasodilators. Abbreviations: mPAP, mean pulmonary artery pressure; PA, pulmonary artery; Ao, aorta; sPAP, systolic pulmonary artery pressure. Other abbreviations are defined in Figure 1.

**FIGURE 5:**
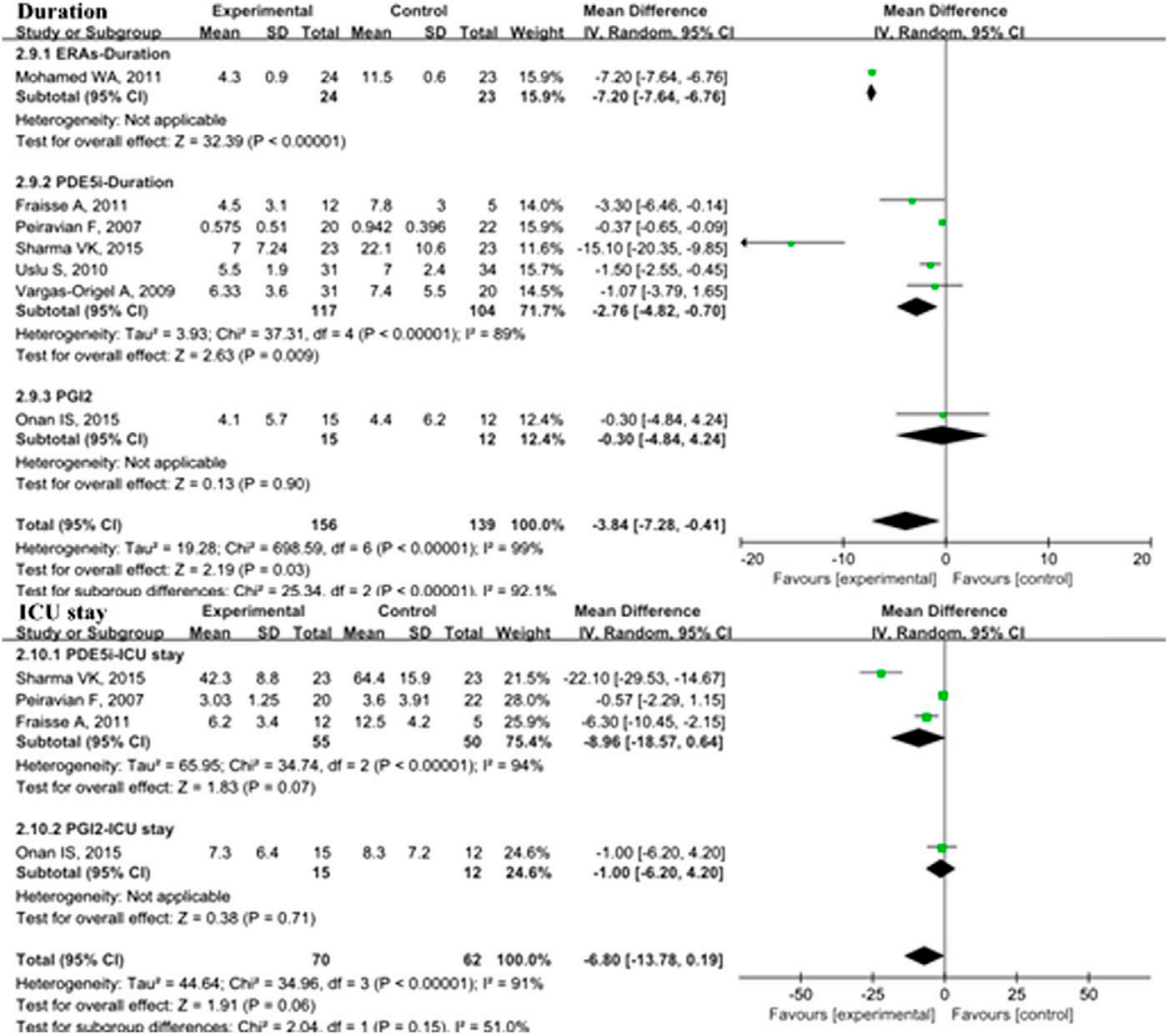
Duration of mechanical ventilation and ICU stay in pediatric PH patients treated with pulmonary vasodilators. Abbreviations: Duration, duration of mechanical ventilation; ICU, intensive care unit. Other abbreviations are defined in Figure 1.

In the overall analysis of pediatric PH patients, OI (MD: −13.34; 95% CI: −23.89 to −2.80; *p* = 0.01; I^2^ = 98%; 5 trials with 176 patients; Figure 3), mPAP (MD: −3.35 mmHg; 95% CI: −6.24 to −0.47; *p* = 0.02; I^2^ = 58%; 8 trials with 462 patients; Figure 4), sPAP (MD: −11.19 mmHg; 95% CI: −16.50 to −5.88; *p* < 0.0001; I^2^ = 0%; 2 trials with 59 patients; Figure 4), PA/AO (MD: −0.11; 95% CI: −0.17 to −0.04; *p* < 0.0008; I^2^ = 53%; 4 trials with 137 patients; Figure 4), and mechanical ventilation duration (MD: −3.84 days; 95% CI: −7.28 to −0.41; *p* = 0.03; I^2^ = 99%; 7 trials with 295 patients; Figure 5) were significantly lower and PaO_2_ (MD: −8.42 mmHg; 95% CI: −11.88 to −4.96; *p* < 0.00001; *I* ^2^ = 0%; 3 trials with 84 patients; Figure 2) was markedly higher in the pulmonary vasodilator group than in the control group. The analysis showed no significant differences in SpO_2_ change (*p* = 0.13; 4 trials with 105 patients; Figure 2) and ICU stay (*p* = 0.06; 4 trials with 132 patients; Figure 5) between the pulmonary vasodilator and control groups.

This analysis showed no heterogeneity in mortality, adverse events, sPAP, and PaO_2_ (I^2^ < 25%); moderate heterogeneity in mPAP and PA/AO (50% < *I* ^2^ < 75%); and high heterogeneity in OI and mechanical ventilation duration (I^2^ > 75%). Through sensitivity analysis, after removing the PGI_2_ subgroup, the heterogeneities of mPAP and PA/AO were reduced to the low level (MD: −4.49, *p* < 0.0001, I^2^ = 11%; MD: −0.12, *p* < 0.00001, I^2^ = 0%; respectively). After removing the PDE5i subgroup, the heterogeneity of OI was reduced to a moderate level but no statistical significance (MD: 0.38, *p* = 0.41, I^2^ = 74). The heterogeneity of mechanical ventilation duration was decreased to 87% after removing the ERAs subgroup but still a high level.

### Subgroup Analysis of Pulmonary Vasodilators in Pediatric PH

#### Primary Outcomes

In the PDE5i subgroup, the incidence of mortality in pediatric PH patients treated with pulmonary vasodilators was significantly lower than that in the control group (RR: 0.20; 95% CI: 0.06 to 0.64; *p* = 0.007; *n* = 522; I^2^ = 0%; Figure 1), equating to an absolute risk difference of −0.05 (95% CI, −0.12 to 0.02). In the ERA subgroup, the mortality was not significantly different compared with the control group (*n* = 112). There were no deaths in the PGI_2_ subgroup (*n* = 69). Subgroup analyses comparing PDE5i, ERAs, and PGI_2_ with the control group treatment for pediatric PH showed no significant differences in adverse events.

#### Secondary Outcomes

In the subgroup analysis of the PDE5i group compared with the control, OI (MD: −19.05; 95% CI: −32.83 to −5.27; *p* = 0.007; I^2^ = 96%; *n* = 102; Figure 2), mPAP (MD: −4.71 mmHg; 95% CI: −7.75 to −1.67; *p* = 0.002; *n* = 333; I^2^ = 33%; Figure 4), sPAP (MD: −11.19 mmHg; 95% CI: −16.50 to −5.88; *p* < 0.0001; I^2^ = 0%; *n* = 59; Figure 4), PA/AO (*p* < 0.00001; *n* = 88; I^2^ = 0%; Figure 4), and mechanical ventilation duration (MD: −2.76 days; 95% CI: −4.82 to −0.70; *p* = 0.009; *n* = 221; I^2^ = 89%; Figure 5) were significantly decreased and PaO_2_ was increased (*p* = 0.03; I_2_ = 36%; *n* = 57; Figure 2). The ERA subgroup analysis showed that the OI (*p* = 0.04; *n* = 47; Figure 2), mPAP (*p* = 0.006; *n* = 60; Figure 4), and mechanical ventilation duration (*p* < 0.00001; *n* = 47; Figure 5) was significantly lower than in the control group. PaO_2_, sPAP, and PA/AO were not reported for the ERA group. In the PGI_2_ subgroup, compared with the control group, PaO_2_ was significantly improved (*p* = 0.001; *n* = 27; Figure 2) and there was no significant difference in OI (*p* = 0.19), mPAP (*p* = 0.82), PA/AO (*p* = 0.34), or mechanical ventilation duration (*p* = 0.90). Indicators of sPAP were not reported for the PGI_2_ subgroup. All subgroup analyses of the pulmonary vasodilators compared with the control group treatments showed no significant changes in SpO_2_ and ICU stay.

### Subgroup Analysis of Pediatric PH With Different Pulmonary Vasodilators

#### Primary Outcomes

Five studies comparing PDE5i in PPHN patients (*n* = 183) showed significantly lower mortality in the vasodilator group (RR: 0.23; 95% CI: 0.06 to 0.83; *p* = 0.03; I^2^ = 0%; Supplementary Table S5 ) than in the control group, equating to an absolute risk difference of −0.11 (95% CI, −0.25 to 0.04). There was no death due to POPH (*n* = 69) in either the PGI_2_ group or the control group. Subgroup analyses of PPHN, POPH, and PPAH compared the treatment group with the control group, and none showed significant differences in adverse events.

#### Secondary Outcomes

The analysis of PPHN patient subgroups showed significantly lower OI with PDE5i (MD: −19.05; 95% CI: −32.83 to −5.27; *p* = 0.007; I^2^ = 96%; *n* = 102) and ERA treatment (*p* = 0.04; *n* = 47), shorter mechanical ventilation duration with PDE5i (MD: −1.44 days; 95% CI: −2.42 to −0.47; *p* = 0.004; I^2^ = 0%; *n* = 116) and ERA treatment (*p* < 0.00001; *n* = 47), and increased PaO_2_ with PDE5i treatment (*p* = 0.03; I^2^ = 36%; *n* = 57) than in the control treatment groups (S5 Table 2). Only one study with 60 PPAH patients showed that mPAP was significantly lower in the group treated with ERAs than in the control group (*p* = 0.006). In POPH patients, subgroup analyses showed that, compared with the placebo group, a significant improvement in PaO_2_ was observed with PGI_2_ treatment (*p* = 0.001; *n* = 27), sPAP with PDE5i (MD: −11.19; 95% CI: −16.50 to −5.88; *p* < 0.0001; *n* = 59), and PA/AO with PDE5i (*p* < 0.00001; I^2^ = 0%; *n* = 88).

### Follow-Up Duration Subgroup Analysis

#### Primary Outcomes

There were four studies with 147 short-term follow-up patients, three studies with 66 mid-term follow-up patients, and eight studies with 506 patients with long-term follow-up duration. In the long-term subgroup analysis, the mortality of pediatric PH patients with pulmonary vasodilators was significantly decreased comparing with the control group (RR: 0.25; 95% CI: 0.07 to 0.82; *p* = 0.02; I^2^ = 0%; Supplementary Table S6), equating to an absolute risk difference of −0.03 (95% CI, −0.10 to 0.03). However, in short-term and mid-term subgroups, the difference in mortality was not significant between the pulmonary vasodilators group and the control group (*p* = 0.15, *p* = 0.10, respectively). The adverse events did not differ between the pulmonary vasodilators group and the control group in the follow-up duration subgroup analyses.

#### Secondary Outcomes

In the short-term subgroup, PA/AO pressure (*p* < 0.00001; S6 Table 3) and OI (*p* < 0.00001) were significantly decreased in the pulmonary vasodilators group. The improvements of mPAP (*p* = 0.03) and PaO_2_ (*p* = 0.01) were significant comparing the pulmonary vasodilators group with the control in the mid-term follow-up subgroup. The analysis in long-term subgroup showed that pulmonary vasodilators significantly decreased mPAP (*p* = 0.0001), PA/AO pressure (*p* = 0.0007), duration of mechanical ventilation (*p* = 0.004), and ICU stay (*p* < 0.00001).

## Discussion

This is the first meta-analysis on the efficacy and safety of pulmonary vasodilators in pediatric PH with subgroups of pulmonary vasodilators and subgroups of patients. The present study demonstrates the clinical benefits of pulmonary vasodilators for the treatment of pediatric PH, including decreased mortality, improved hemodynamics (OI, PaO_2_, mPAP, sPAP, and PA/Ao), and shorter duration of mechanical ventilation compared with those of the control group.

Three pulmonary vasodilators were used to treat pediatric PH in the present study: PDE5i (sildenafil), ERAs (bosentan), and PGI_2_ (Iloprost or Beraprost). Sildenafil is a highly selective PDE5i that can enhance the concentration of cyclic guanosine monophosphate in pulmonary vascular smooth muscle cells by inhibiting PDE5 and promoting endogenous nitric oxide function (Archer and Michelakis, 2009). Sildenafil can also alleviate pulmonary vascular remodeling by inhibiting the proliferation of vascular smooth muscle cells (Layton and Bavdekar, 2015). Bosentan is a nonselective ERA with an affinity for ETA and ETB that can dilate pulmonary blood vessels (Barst et al., 2003). Iloprost and beraprost are analogs of PGI_2_, which can selectively dilate pulmonary blood vessels and inhibit proliferation and thrombotic effects (Cheng et al., 2002). All three categories of pulmonary vasodilators showed beneficial effects in pediatric PH patients. Sildenafil therapy showed a significant improvement in mortality in pediatric PH patients, but mortality in patients treated with ERAs and PGI_2_ was not significantly different from that in the control group. Besides, no deaths occurred in the PGI_2_ studies during follow-up, which also demonstrates the safety of PGI_2_. Compared with the previous studies in adults, adverse events in the present study for sildenafil were headache (34% in adult vs. 13% in pediatric patients), gastrointestinal upset (26% in adult vs. 11% in pediatric patients), respiratory symptoms (11% in adult vs. 12% in pediatric patients), and epistaxis (6% in adult vs. 3% in pediatric patients) (Peiravian et al., 2007; Cornelisse et al., 2012; Barnes et al., 2019); the adverse events for bosentan were headache (21% in adult vs. 0% in pediatric patients), respiratory symptoms (8% in adult vs. 9% in pediatric patients), abnormal hepatic function (9% in adult vs. 0% in pediatric patients), red blood cell transfusion (0% in adult vs. 31% in pediatric patients), and anemia (0% in adult vs. 23% in pediatric patients) (Rubin et al., 2002; Gibbs and Ismail, 2012; Steinhorn et al., 2016); and the adverse events for beraprost sodium and iloprost treatment were headache (53% in adult vs. 0% in pediatric patients), upper respiratory tract events (34% in adult vs. 0% in pediatric patients), nausea or vomiting (43% in adult vs. 0% in pediatric patients), PH crisis (6% in adult vs. 7% in pediatric patients), and thrombocytopenia (0% in adult vs. 27% in pediatric patients) (Xu et al., 2015; Onan et al., 2016; Pulido et al., 2019). All pediatric PH patients well tolerated the pulmonary vasodilators.

**FIGURE 6:**
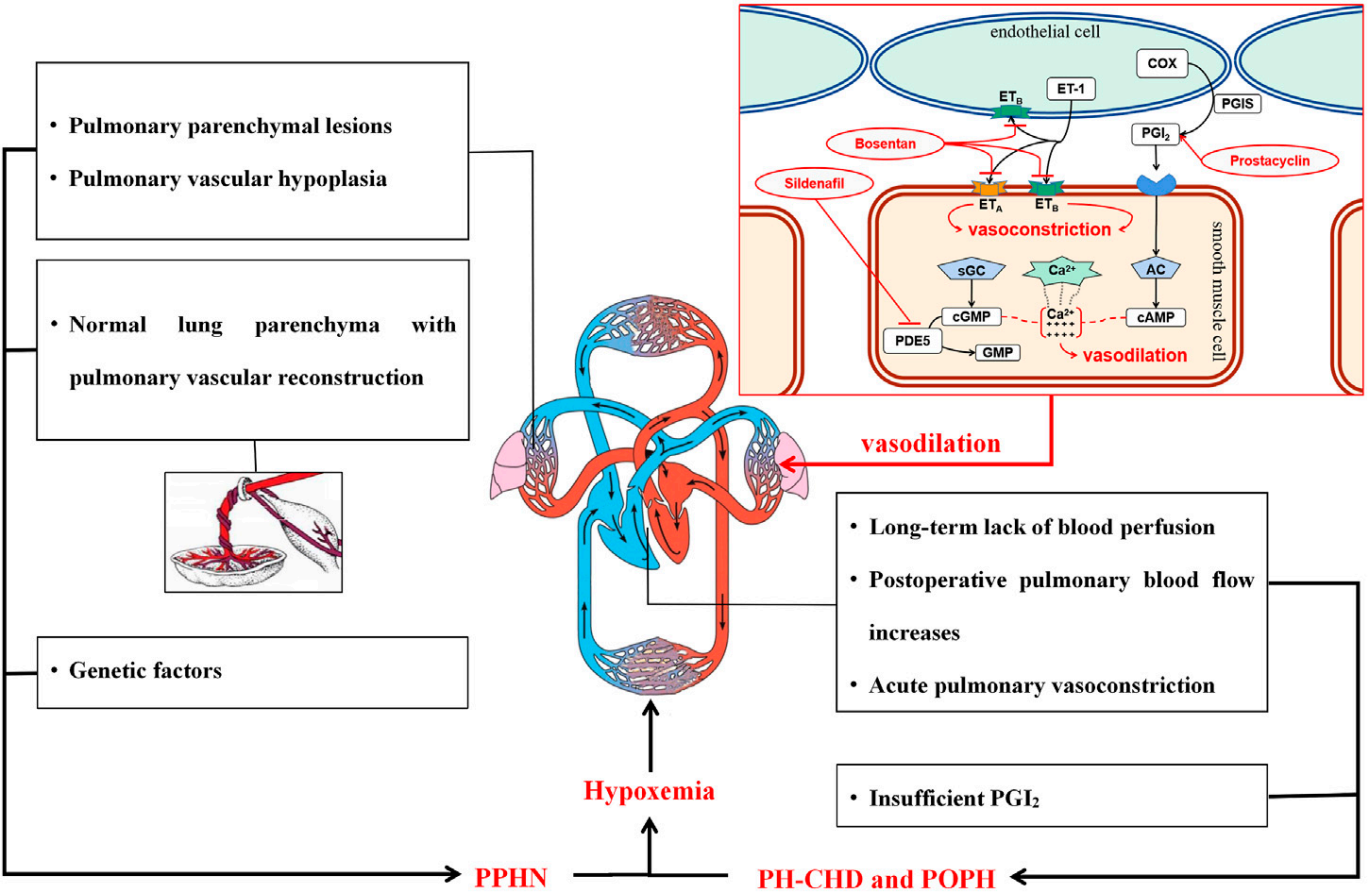
Pathological mechanisms of pediatric PH and pulmonary vasodilators. Abbreviations: PVR, pulmonary vascular resistance; SVR, systemic vascular resistance; ASD, atrial septal defect; VSD, ventricular septal defect; PDA, patent ductus arteriosus; ET-1, endothelin-1; ETA, endothelin receptor A; ETB, endothelin receptor B; PDE5, type 5 phosphodiesterase; COX, cyclooxygenase; PGIS, prostacyclin synthase; PGI2, prostacyclin; sGC, soluble guanylate cyclase; cGMP, cyclic guanosine monophosphate; GMP, guanosine monophosphate; AC, adenylate cyclase; cAMP, 3′-5′ cyclic adenosine monophosphate.

The pathological mechanisms of pediatric PH are shown in Figure 6. PPHN is a persistent increase in the PVR after birth, and the fetal-type circulation cannot transition normally to the adult-type circulation, resulting in unoxygenated blood flow shunting from the ovale or arterial catheter from the right to the left side of the heart, causing hypoxemia (Puthiyachirakkal and Mhanna, 2013; Teng and Wu, 2017). Lung parenchymal injury and dysplasia caused by meconium aspiration and lung infection are the most common causes of PPHN. The main manifestations of pulmonary vascular hypoplasia include abnormal conduction response (García-Cardeña et al., 1996), abnormal development, and microthrombosis (Rabinovitch et al., 1987) in the pulmonary vascular bed. Normal lung parenchyma with pulmonary vascular reconstruction is partially due to intrauterine factors, including chronic hypoxia of the fetus caused by oligohydramnios (Olley et al., 1981), vasoactive drugs crossing from the placental circulation to the fetal pulmonary circulation (Koren and Nordeng, 2012; 't Jong et al., 2012; Van Marter et al., 2013), and occlusion of the intrauterine artery (Pearson et al., 2001). Genetic factors are also involved in the development of PPHN. The pathological mechanisms of PH-CHD and POPH mainly include lack of blood perfusion, increased blood flow after the operation, acute pulmonary vasoconstriction, and altered PGI_2_ signaling (Wang et al., 2005; Scott, 2010; Taylor and Laussen, 2010). The present study found that PDE5i treatment improved mortality in patients with PPHN. Although ERA treatment did not significantly change the mortality of patients with PPHN, it significantly improved the respiratory and hemodynamic parameters in the patients. This may be related to the pathogenesis of PPHN. The pulmonary vessels of PPHN patients were not completely irreversibly changed. By dilating the pulmonary blood vessels, the vascular hypoxic state may be corrected. For patients with POPH, neither PDE5i nor PGI_2_ significantly reduced mortality, but they significantly improved respiratory and hemodynamic parameters. This result may be related to the reconstruction of pulmonary blood vessels in patients with POPH before surgery. The increased blood flow in the pulmonary circulation after the repair surgery may further harm pulmonary vessels, but the pulmonary vasodilators allow the blood to flow with less resistance, significantly improving clinical symptoms. The fact that the mortality rate does not decrease significantly may also be related to the short duration of follow-up time and insufficient patients in the included studies.

We used GRADEpro GDT software to score the quality of evidence (Supplementary Table S6). The research evidence is inaccurate due to the small number of studies included and the small number of participants. The substances used in the control groups were not completely uniform; some studies used distilled water, MgSO_4_, or tolazoline, and some studies did not explicitly describe the ingredients of the placebo. The measurement of some outcome indicators lacks a unified method and standard.

There were some limitations of the study. First, the meta-analysis included limited number of clinical trials and small sample sizes. Therefore, extra caution should be exercised when interpreting the treatment efficacy for the outcomes. Second, the studies in which the diagnosis of PH based on echocardiography were included. That is not the golden definition of PAH and may weaken the results. Third, some of the included studies have not evaluated the clinical endpoints, such as sPAP, mPAP, etc. Therefore, it may lead to limited and biased results. The original study lacked some key hemodynamic parameters such as PVR, CI, and CO. Most pediatric patients <15 years of age require conscious sedation or general anesthesia during cardiac catheterization which may lead to instability with induction and adverse effects, such as hypoxia and hypercapnia. During or after a cardiac catheterization, pediatrics with PH may experience acute deterioration. The possible reason may be that the parents of the pediatric patients might reject the invasive catheterization due to the potential risk. Therefore, these effect indicators are not included in the outcomes of our research. In addition, the potential effects of different ages on the response to pulmonary vasodilators in the cohort of patients with pediatric PAH may interfere with the statistical power of the conclusions. However, due to the limited number and size of included studies, an age subgroups analysis was difficult to be performed.

## Conclusion

The present study shows that pulmonary vasodilators decrease mortality, improve respiratory and hemodynamic parameters, and shorten the duration of mechanical ventilation in pediatric PH patients. In PPHN, PDE5i significantly decrease mortality, and ERAs improve respiratory and hemodynamic parameters. In POPH, respiratory and hemodynamic indicators are markedly improved with PDE5i. The long-term benefits of pulmonary vasodilators for patients with pediatric PH are more clear. Overall, pulmonary vasodilators were well tolerated.

## Data Availability

The original contributions presented in the study are included in the article/Supplementary Material, further inquiries can be directed to the corresponding author.
