# Supplementary material for: The Efficacy and Safety of Pulmonary Vasodilators in Pediatric Pulmonary Hypertension (PH): A Systematic Review and Meta-analysis
Source: Front Pharmacol. 2021 Apr 23;12:668902. doi: 10.3389/fphar.2021.668902 (PMC8103162; doi:10.3389/fphar.2021.668902)
Supplement: Supplementary file 1 [file DataSheet1.docx]

**Supplements**

**S1 Table 1.** Search expression in each database.

| **Database** | **Search formula** |
| --- | --- |
| Pubmed  (338 results) | ((((((Pediatric pulmonary hypertension) OR Pediatric pulmonary artery hypertension) OR "Pediatric pulmonary hypertension"[Mesh]) OR "Pediatric pulmonary hypertension"[Mesh])) AND (((((((((((drug therapy) OR "Drug Therapy"[Mesh]) OR specific therapy) OR "Molecular Targeted Therapy"[Mesh])) OR Pulmonary vasodilators) OR ((((((((Endothelin receptor antagonists) OR "Endothelin Receptor Antagonists"[Mesh]) OR Ambrisentan) OR "ambrisentan" [Supplementary Concept]) OR Bosentan) OR "Bosentan"[Mesh]) OR Macitentan) OR "macitentan" [Supplementary Concept])) OR ((((((((Phosphodiesterase type 5 inhibitors) OR "Phosphodiesterase 5 Inhibitors"[Mesh]) OR Sildenafil) OR "Sildenafil Citrate"[Mesh]) OR Tadalafil) OR "Tadalafil"[Mesh]) OR Vardenafil) OR "Vardenafil Dihydrochloride"[Mesh])) OR (((Soluble guanylate cyclase stimulators) OR riociguat) OR "riociguat" [Supplementary Concept])) OR ((((((((((((Prostacyclin analogues) OR Prostacyclin) OR "benzodioxane prostacyclin" [Supplementary Concept]) OR Beraprost) OR "beraprost" [Supplementary Concept]) OR Epoprostenol) OR "Epoprostenol"[Mesh]) OR synthetic prostacyclin) OR Iloprost) OR "Iloprost"[Mesh]) OR Treprostinil) OR "treprostinil" [Supplementary Concept])) OR (((prostacyclin receptor agonists) OR Selexipag) OR "selexipag" [Supplementary Concept]))) AND ((random*) OR (("Randomized Controlled Trial" [Publication Type]) OR "Randomized Controlled Trials as Topic" [Mesh])) |
| Embase  (102 results) | ('pediatric pulmonary artery hypertension' OR 'pediatric pulmonary hypertension') AND ('drug therapy' OR 'drug therapy' OR 'specific therapy' OR 'pulmonary vasodilators' OR 'endothelin receptor antagonists' OR 'ambrisentan' OR 'bosentan' OR 'macitentan' OR 'phosphodiesterase inhibitor 5' OR 'phosphodiesterase v inhibitor' OR 'sildenafil' OR 'tadalafil' OR 'vardenafil' OR 'soluble guanylate cyclase stimulators' OR 'riociguat' OR 'prostacyclin analogues' OR 'prostacyclin' OR 'beraprost' OR 'epoprostenol' OR 'prostacyclin derivative' OR 'synthetic prostacyclin' OR 'iloprost' OR 'treprostinil' OR 'prostacyclin receptor agonists' OR 'selexipag') AND (random* OR 'randomized controlled trials') |
| Cochrane Library  (34 results) | (pediatric pulmonary artery hypertension or pediatric pulmonary hypertension) AND (drug Therapy OR Endothelin receptor antagonists OR bosentan OR phosphodiesterase 5 Inhibitors or sildenafil Citrate OR tadalafil OR Vardenafil Dihydrochloride OR Prostaglandins I OR Epoprostenol OR Iloprost) AND Randomized Controlled Trial |

**S2 Figure 1.** Flow chart of study selection.


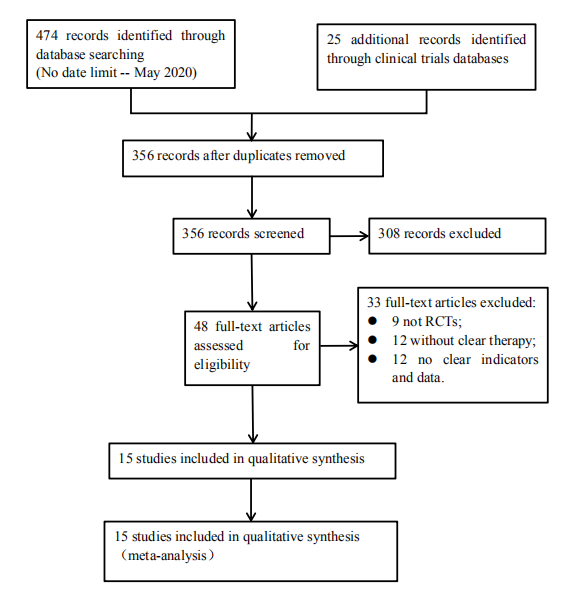


**S3 Figure 2.** The details of the risk of bias in the included studies.


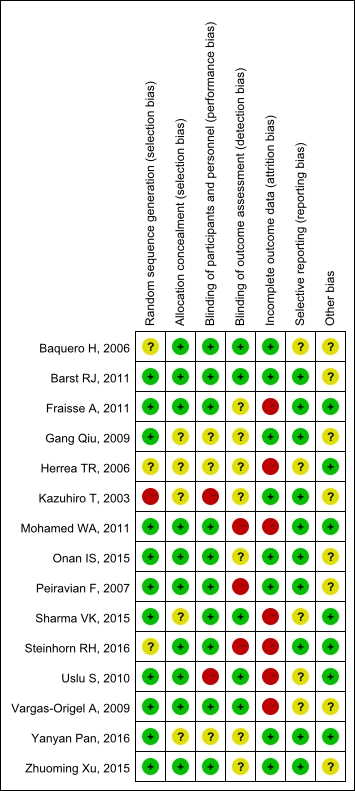


**S4 Figure 3.** A summary of the risk of bias in the included studies.


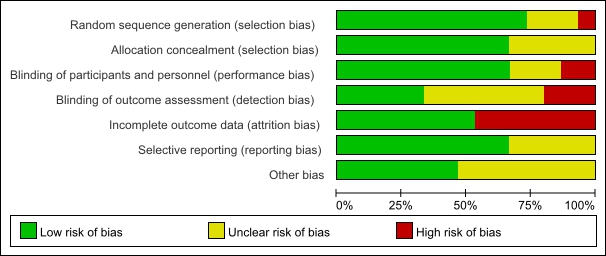


**S5 Table 2.** Subgroup analysis of pulmonary vasodilators in different types of pediatric pulmonary hypertension patients.

| **PDE5i-PPHN** | | | | | | | | | | | | | |
| --- | --- | --- | --- | --- | --- | --- | --- | --- | --- | --- | --- | --- | --- |
| Primary Outcomes | | References | E (Events) | | E (Total) | C (Events) | C (Total) | | RR | 95%CI | | P | I^2^ (%) |
| Death | | 5 | 2 | | 97 | 10 | 86 | | 0.23 | 0.06 to 0.83 | | 0.03 | 0 |
| Adverse events | | 2 | 0 | | 46 | 0 | 49 | | NE | NE | | NA | NA |
| Secondary Outcomes | | References | E (Total) | |  | C (Total) |  | | MD | 95%CI | | P | I^2^ (%) |
| mPAP, mmHg | | 2 | 46 | |  | 49 |  | | -4.43 | -9.14 to 0.27 | | 0.06 | 65 |
| sPAP, mmHg | | 0 | - | |  | - |  | | - | - | | - | - |
| PA/Ao pressure | | 0 | - | |  | - |  | | - | - | | - | - |
| OI | | 3 | 51 | |  | 51 |  | | -19.05 | -32.83 to -5.27 | | 0.007 | 96 |
| PaO_2_, mmHg | | 2 | 30 | |  | 27 |  | | -7.42 | -14.05 to -0.79 | | 0.03 | 36 |
| SpO_2_, % | | 2 | 22 | |  | 21 |  | | 10.32 | -7.51 to 28.15 | | 0.26 | 94 |
| Duration of mechanical ventilation, days | | 2 | 62 | |  | 54 |  | | -1.44 | -2.42 to -0.47 | | 0.004 | 0 |
| ICU stay, days | | 0 | - | |  | - |  | | - | - | | - | - |
| **PDE5i-POPH** | | | | | | | | | | | | | |
| Primary Outcomes | | References | E (Events) | | E (Total) | C (Events) | C (Total) | | RR | 95%CI | | P | I^2^ (%) |
| Death | | 3 | 0 | | 55 | 2 | 50 | | 0.09 | 0.01 to 1.64 | | 0.1 | NA |
| Adverse events | | 3 | 3 | | 55 | 7 | 50 | | 0.55 | 0.17 to 1.79 | | 0.32 | 0 |
| Secondary Outcomes | | References | E (Total) | |  | C (Total) |  | | MD | 95%CI | | P | I^2^ (%) |
| mPAP, mmHg | | 1 | 12 | |  | 5 |  | | -13.00 | -26.45 to 0.45 | | 0.06 | NA |
| sPAP, mmHg | | 2 | 32 | |  | 27 |  | | -11.19 | -16.50 to -5.88 | | <0.0001 | 0 |
| PA/Ao pressure | | 2 | 43 | |  | 45 |  | | -0.12 | -0.16 to -0.08 | | < 0.00001 | 0 |
| OI | | 0 | - | |  | - |  | | - | - | | - | - |
| PaO_2_, mmHg | | 0 | - | |  | - |  | | - | - | | - | - |
| SpO_2_, % | | 0 | - | |  | - |  | | - | - | | - | - |
| Duration of mechanical ventilation, days | | 3 | 55 | |  | 50 |  | | -5.72 | -12.24 to 0.80 | | 0.09 | 94 |
| ICU stay, days | | 3 | 55 | |  | 50 |  | | -8.96 | -18.57 to 0.64 | | 0.07 | 94 |
| **PDE5i-PPAH** |  | | |  | | | |  | | |  | | |
| Primary Outcomes | | References | E (Events) | | E (Total) | C (Events) | C (Total) | | RR | 95%CI | | P | I^2^ (%) |
| Death | | 1 | 0 | | 174 | 0 | 60 | | NE | NE | | NA | NA |
| Adverse events | | 1 | 9 | | 174 | 2 | 60 | | 1.55 | 0.34 to 6.98 | | 0.57 | NA |
| Secondary Outcomes | | References | E (Total) | |  | C (Total) |  | | MD | 95%CI | | P | I^2^ (%) |
| mPAP, mmHg | | 1 | 165 | |  | 56 |  | | -3.90 | -8.57 to 0.77 | | 0.10 | NA |
| sPAP, mmHg | | 0 | - | |  | - |  | | - | - | | - | - |
| PA/Ao pressure | | 0 | - | |  | - |  | | - | - | | - | - |
| OI | | 0 | - | |  | - |  | | - | - | | - | - |
| PaO_2_, mmHg | | 0 | - | |  | - |  | | - | - | | - | - |
| SpO_2_, % | | 0 | - | |  | - |  | | - | - | | - | - |
| Duration of mechanical ventilation, days | | 0 | - | |  | - |  | | - | - | | - | - |
| ICU stay, days | | 0 | - | |  | - |  | | - | - | | - | - |
| **ERAs-PPHN** |  | | |  | | | |  | | |  | | |
| Primary Outcomes | | References | E (Events) | | E (Total) | C (Events) | C (Total) | | RR | 95%CI | | P | I^2^ (%) |
| Death | | 2 | 1 | | 30 | 3 | 22 | | 0.20 | 0.02 to 1.77 | | 0.15 | NA |
| Adverse events | | 2 | 6 | | 36 | 10 | 22 | | 0.40 | 0.08 to 1.86 | | 0.24 | 64 |
| Secondary Outcomes | | References | E (Total) | |  | C (Total) |  | | MD | 95%CI | | P | I^2^ (%) |
| mPAP, mmHg | | 0 | - | |  | - |  | | - | - | | - | - |
| sPAP, mmHg | | 0 | - | |  | - |  | | - | - | | - | - |
| PA/Ao pressure | | 0 | - | |  | - |  | | - | - | | - | - |
| OI | | 1 | 24 | |  | 23 |  | | -9.80 | -19.35 to -0.25 | | 0.04 | NA |
| PaO_2_, mmHg | | 0 | - | |  | - |  | | - | - | | - | - |
| SpO_2_, % | | 1 | 24 | |  | 23 |  | | 11.08 | 0.30 to 21.86 | | 0.04 | NA |
| Duration of mechanical ventilation, days | | 1 | 24 | |  | 23 |  | | -7.20 | -7.64 to -6.76 | | <0.00001 | NA |
| ICU stay, days | | 0 | - | |  | - |  | | - | - | | - | - |
| **ERAs-PPAH** |  | | |  | | | |  | | |  | | |
| Primary Outcomes | | References | E (Events) | | E (Total) | C (Events) | C (Total) | | RR | 95%CI | | P | I^2^ (%) |
| Death | | 1 | 0 | | 30 | 0 | 30 | | NE | NE | | NA | NA |
| Adverse events | | 1 | 0 | | 30 | 0 | 30 | | NE | NE | | NA | NA |
| Secondary Outcomes | | References | E (Total) | |  | C (Total) |  | | MD | 95%CI | | P | I^2^ (%) |
| mPAP, mmHg | | 1 | 30 | |  | 30 |  | | -4.25 | -7.26 to -1.24 | | 0.006 | NA |
| sPAP, mmHg | | 0 | - | |  | - |  | | - | - | | - | - |
| PA/Ao pressure | | 0 | - | |  | - |  | | - | - | | - | - |
| OI | | 0 | - | |  | - |  | | - | - | | - | - |
| PaO_2_, mmHg | | 0 | - | |  | - |  | | - | - | | - | - |
| SpO_2_, % | | 0 | - | |  | - |  | | - | - | | - | - |
| Duration of mechanical ventilation, days | | 0 | - | |  | - |  | | - | - | | - | - |
| ICU stay, days | | 0 | - | |  | - |  | | - | - | | - | - |
| **PGI_2_-POPH** |  | | |  | | | |  | | |  | | |
| Primary Outcomes | | References | E (Events) | | E (Total) | C (Events) | C (Total) | | RR | 95%CI | | P | I^2^ (%) |
| Death | | 3 | 0 | | 37 | 0 | 32 | | NE | NE | | NA | NA |
| Adverse events | | 2 | 8 | | 30 | 6 | 19 | | 0.77 | 0.23 to 2.58 | | 0.67 | 44 |
| Secondary Outcomes | | References | E (Total) | |  | C (Total) |  | | MD | 95%CI | | P | I^2^ (%) |
| mPAP, mmHg | | 3 | 37 | |  | 32 |  | | -0.89 | -8.60 to 6.81 | | 0.82 | 64 |
| sPAP, mmHg | | 0 | - | |  | - |  | | - | - | | - | - |
| PA/Ao pressure | | 2 | 30 | |  | 19 |  | | -0.13 | -0.39 to 0.13 | | 0.34 | 80 |
| OI | | 1 | 15 | |  | 15 |  | | -0.30 | -0.75 to 0.15 | | 0.19 | NA |
| PaO_2_, mmHg | | 1 | 15 | |  | 12 |  | | -8.70 | -13.90 to -3.50 | | 0.001 | NA |
| SpO_2_, % | | 1 | 7 | |  | 8 |  | | -1.20 | -6.77 to 4.37 | | 0.67 | NA |
| Duration of mechanical ventilation, days | | 1 | 15 | |  | 12 |  | | -0.30 | -4.84 to 4.24 | | 0.90 | NA |
| ICU stay, days | | 1 | 15 | |  | 12 |  | | -1.00 | -6.20 to 4.20 | | 0.71 | NA |

RR: Risk Ratio; CI: confidence interval; MD: Mean Difference; NE: Not Estimable; NA: Not Applicable; mPAP: mean pulmonary artery pressure; sPAP: systolic pulmonary artery pressure; PA: pulmonary artery; Ao: aorta; OI: oxygenation index; PaO2: partial pressure of arterial oxygen; SpO2: pulse oxygen saturation; ICU: Intensive Care Unit. Other abbreviations are declared as Table 1 - 2.

**S6 Table 3.** Subgroup analysis of short and long follow-up duration in pediatric pulmonary hypertension patients with pulmonary vasodilators.

| **Follow-up duration** | | | | | | | | | |
| --- | --- | --- | --- | --- | --- | --- | --- | --- | --- |
| Primary Outcomes | References | E (Events) | E (Total) | C (Events) | C (Total) | RR | 95%CI | P | I^2^ (%) |
| Death | | | | | | | | | |
| Short-term | 4 | 0 | 79 | 3 | 68 | 0.12 | [0.01, 2.14] | 0.15 | NA |
| Mid-term | 3 | 0 | 42 | 2 | 24 | 0.09 | [0.01, 1.64] | 0.10 | NA |
| Long-term | 8 | 3 | 302 | 10 | 188 | 0.25 | [0.07, 0.82] | 0.02 | 0 |
| Adverse events | | | | | | | | | |
| Short-term | 2 | 3 | 35 | 5 | 37 | 0.66 | [0.18, 2.41] | 0.53 | NA |
| Mid-term | 3 | 8 | 42 | 6 | 24 | 0.77 | [0.23, 2.58] | 0.67 | 44 |
| Long-term | 6 | 15 | 294 | 14 | 169 | 0.54 | [0.19, 1.55] | 0.25 | 42 |
| Secondary Outcomes | References | E (Total) |  | C (Total) |  | MD | 95%CI | P | I^2^ (%) |
| mPAP, mmHg | | | | | | | | | |
| Short-term | 1 | 15 |  | 15 |  | -2.00 | [-6.00, 2.00] | 0.33 | NA |
| Mid-term | 1 | 27 |  | 24 |  | -3.57 | [-19.46, 12.32] | 0.03 | 80 |
| Long-term | 4 | 233 |  | 133 |  | -4.37 | [-6.59, -2.15] | 0.0001 | 15 |
| sPAP, mmHg | | | | | | | | | |
| Short-term | 1 | 20 |  | 22 |  | -10.80 | [-16.46, -5.14] | 0.0002 | NA |
| Mid-term | 1 | 12 |  | 5 |  | -14.00 | [-29.28, 1.28] | 0.07 | NA |
| Long-term | 0 | - |  | - |  | - | - | - | - |
| PA/Ao pressure | | | | | | | | | |
| Short-term | 1 | 20 |  | 22 |  | -0.13 | [-0.18, -0.08] | < 0.00001 | NA |
| Mid-term | 2 | 30 |  | 19 |  | -0.13 | [-0.39, 0.13] | 0.34 | 80 |
| Long-term | 1 | 23 |  | 23 |  | -0.10 | [-0.16, -0.04] | 0.0007 | NA |
| OI | | | | | | | | | |
| Short-term | 1 | 13 |  | 11 |  | -19.50 | [-23.31, -15.69] | < 0.00001 | NA |
| Mid-term | 1 | 15 |  | 12 |  | -0.30 | [-0.75, 0.15] | 0.19 | NA |
| Long-term | 3 | 62 |  | 63 |  | -16.08 | [-33.62, 1.46] | 0.07 | 94 |
| PaO_2_, mmHg | | | | | | | | | |
| Short-term | 2 | 28 |  | 26 |  | 9.66 | [-13.24, 32.56] | 0.41 | 93 |
| Mid-term | 1 | 15 |  | 12 |  | -8.70 | [-13.90, -3.50] | 0.001 | NA |
| Long-term | 0 | - |  | - |  | - | - | - | - |
| SpO_2_, % | | | | | | | | | |
| Short-term | 1 | 15 |  | 15 |  | 1.40 | [-3.40, 6.20] | 0.57 | NA |
| Mid-term | 0 | - |  | - |  | - | - | - | - |
| Long-term | 3 | 38 |  | 37 |  | 9.66 | [-4.42, 23.75] | 0.18 | 91 |
| Duration of mechanical ventilation, days | | | | | | | | | |
| Short-term | 2 | 51 |  | 42 |  | -4.32 | [-11.80, 3.16] | 0.26 | 78 |
| Mid-term | 2 | 27 |  | 17 |  | -2.26 | [-5.06, 0.54] | 0.11 | 11 |
| Long-term | 3 | 78 |  | 80 |  | -7.18 | [-12.11, -2.25] | 0.004 | 98 |
| ICU stay, days | | | | | | | | | |
| Short-term | 1 | 20 |  | 22 |  | -0.57 | [-2.29, 1.16] | 0.52 | NA |
| Mid-term | 2 | 27 |  | 17 |  | -3.89 | [-9.06, 1.28] | 0.14 | 59 |
| Long-term | 1 | 23 |  | 23 |  | -22.10 | [-29.53, -14.67] | < 0.00001 | NA |

Abbreviations are declared as S5 Table 2.

**S7 Table 4.** Quality of the evidence.

| **Pulmonary vasodilators compared with placebo for pediatric pulmonary hypertension patients** | | | | | | |
| --- | --- | --- | --- | --- | --- | --- |
| **Patient or population:** patients with pediatric pulmonary hypertension patients **Settings:** Hospital **Intervention:** pulmonary vasodilators | | | | | | |
| **Outcomes** | **Illustrative comparative risks* (95% CI)** | | **Relative effect (95% CI)** | **No of Participants (studies)** | **Quality of the evidence (GRADE)** | **Comments** |
|  | Assumed risk | Corresponding risk |  |  |  |  |
|  | **Control** | **Pulmonary vasodilators** |  |  |  |  |
| **Death** Follow-up: 24 hours -15 months | **Study population** | | **RR 0.2**  (0.07 to 0.56) | 703 (15 studies) | ⊕⊕⊕⊝ **moderate**^1^ | Evidence was downgraded due to indirectness. |
|  | **54 per 1000** | **11 per 1000** (4 to 30) |  |  |  |  |
|  | **Moderate** | |  |  |  |  |
|  | **0 per 1000** | **0 per 1000** (0 to 0) |  |  |  |  |
| **Adverse events** Follow-up: 24 hours -15 months | **Study population** | | **RR 0.63**  (0.35 to 1.12) | 601 (11 studies) | ⊕⊕⊝⊝ **low**^1,2^ | Evidence was downgraded due to indirectness and imprecision. |
|  | **109 per 1000** | **68 per 1000** (38 to 122) |  |  |  |  |
|  | **Moderate** | |  |  |  |  |
|  | **87 per 1000** | **55 per 1000** (30 to 97) |  |  |  |  |
| **OI** Follow-up: 24 hours -15 months |  | The mean oi in the intervention groups was **13.34 lower** (23.89 to 2.8 lower) |  | 176 (5 studies) | ⊕⊕⊝⊝ **low**^3,4^ | Evidence was downgraded due to inconsistency and imprecision. |
| **PaO2** Follow-up: 24 hours -15 months |  | The mean pao2 in the intervention groups was **8.42 lower** (11.88 to 4.96 lower) |  | 84 (3 studies) | ⊕⊕⊕⊝ **moderate**^4^ | Evidence was downgraded due to imprecision. |
| **SpO2** Follow-up: 24 hours -15 months |  | The mean spo2 in the intervention groups was **7.34 higher** (2.09 lower to 16.78 higher) |  | 105 (4 studies) | ⊕⊕⊝⊝ **low**^3,4^ | Evidence was downgraded due to inconsistency and imprecision. |
| **mPAP** Follow-up: 24 hours -15 months |  | The mean mpap in the intervention groups was **3.35 lower** (6.24 to 0.47 lower) |  | 462 (8 studies) | ⊕⊕⊕⊝ **moderate**^5^ | Evidence was downgraded due to indirectness. |
| **PA/Ao** Follow-up: 24 hours -15 months |  | The mean pa/ao in the intervention groups was **0.11 lower** (0.17 to 0.04 lower) |  | 137 (4 studies) | ⊕⊕⊕⊝ **moderate**^5^ | Evidence was downgraded due to indirectness. |
| **sPAP** Follow-up: 24 hours -15 months |  | The mean spap in the intervention groups was **11.19 lower** (16.5 to 5.88 lower) |  | 59 (2 studies) | ⊕⊕⊝⊝ **low**^4,5^ | Evidence was downgraded due to indirectness and imprecision. |
| **Duration of mechanical ventilation** Follow-up: 24 hours -15 months |  | The mean duration of mechanical ventilation in the intervention groups was **3.84 lower** (7.28 to 0.41 lower) |  | 295 (7 studies) | ⊕⊕⊕⊝ **moderate**^3^ | Evidence was downgraded due to inconsistency. |
| **ICU stay** Follow-up: 24 hours -15 months |  | The mean icu stay in the intervention groups was **6.8 lower** (13.78 lower to 0.19 higher) |  | 132 (4 studies) | ⊕⊕⊕⊝ **moderate**^3^ | Evidence was downgraded due to inconsistency. |
| *The basis for the **assumed risk** (e.g. the median control group risk across studies) is provided in footnotes. The **corresponding risk** (and its 95% confidence interval) is based on the assumed risk in the comparison group and the **relative effect** of the intervention (and its 95% CI).  **CI:** Confidence interval; **RR:** Risk ratio; Other abbreviations are declared as Table 2 and 3. | | | | | | |
| GRADE Working Group grades of evidence **High quality:** Further research is very unlikely to change our confidence in the estimate of effect.  **Moderate quality:** Further research is likely to have an important impact on our confidence in the estimate of effect and may change the estimate. **Low quality:** Further research is very likely to have an important impact on our confidence in the estimate of effect and is likely to change the estimate. **Very low quality:** We are very uncertain about the estimate. | | | | | | |
| ^1^ The PICO principles are not completely consistent, and in some studies the control group used different drugs. ^2^ There is no uniform standard for adverse events, and the records of some studies are not detailed enough. ^3^ The I^2 of the five studies more than 75%, P <0.05, with greater heterogeneity. ^4^ The study sample related to this outcome indicator is small. ^5^ The detection methods are not uniform, and some studies are through catheters, and some studies are through cardiac ultrasound. | | | | | | |
